# Sexual Proportion and Egg Hatching of Vector Mosquitos in an Atlantic Forest Fragment in Rio de Janeiro, Brazil

**DOI:** 10.3390/life13010013

**Published:** 2022-12-21

**Authors:** Shayenne Olsson Freitas Silva, Cecilia Ferreira de Mello, Genimar Rebouças Julião, Rayane Dias, Jeronimo Alencar

**Affiliations:** 1 Diptera Laboratory, Oswaldo Cruz Institute (Fiocruz), Avenida Brasil 4365, Manguinhos, Rio de Janeiro 21040-360, RJ, Brazil; 2 Postgraduate Program in Tropical Medicine, Oswaldo Cruz Institute (Fiocruz), Rio de Janeiro 21040-360, RJ, Brazil; 3 Laboratory of Entomology I, Fiocruz Rondônia, Rua da Beira 7671, Lagoa, Porto Velho 76812-245, RO, Brazil; 4 National Institute of Epidemiology of the Western Amazon—INCT EpiAmO—Fiocruz Rondônia, Rua da Beira 7671, Lagoa, Porto Velho 76812-245, RO, Brazil

**Keywords:** Culicidae, vectors, eggs, sex ratio, hatchability

## Abstract

Some Aedinii mosquitoes are of high importance in the transmission of the sylvatic YFV. Usually, their eggs are very resistant and depend on the rain for their hatching. The present study evaluated the effect of multiple mosquito-egg immersions and the sex ratio of male and female specimens from Atlantic Forest remnants in the state of Rio de Janeiro, Brazil. Three sampling sites were selected in the municipality of Casimiro de Abreu, where 50 ovitraps were randomly installed to collect eggs from the ground level up to different heights, from August 2018 to December 2020. The mosquito sex ratios were compared between seasons and forest sites, using the generalized linear mixed model (GLMM), which included sampling months and trees as random effects. A total of 33,091 mosquito eggs were collected, of which 6152 eggs were already hatched (18%) and 26,939 were unhatched; of these, approximately 76% subsequently hatched. We found that 25% of the eggs corresponded to four species: *Aedes albopictus* (n = 1277), *Ae. terrens* (n = 793), *Haemagogus janthinomys* (n = 89), and *Hg. leucocelaenus* (n = 3033). The sex ratio (male:female) was variable concerning the sampling sites and the season. For most species, GLMM estimates found no difference in the variation of the average sex ratio as a function of these predictors, and there was no evidence of temporal autocorrelation in the mosquito data. The number of immersions necessary for hatching the eggs differed between mosquito species, and eggs collected in the dry season hatched both in the first immersions and the subsequent events. Co-occurrence of *Aedes terrens* and *Hg. leucocelaenus* was the most frequently observed pairwise species combination. Considering recurrent arbovirus outbreaks in Brazil and their burden on the human population, our study helps to shed light on how these vectors behave in nature; therefore, they can be used in surveillance programs.

## 1. Introduction

Mosquitoes are responsible for the transmission of several major pathogens, such as the arboviruses that cause Zika, dengue, chikungunya, yellow fever, and the etiological agent of malaria. This matter has drawn attention to these arthropods, as these diseases are considered a serious public-health problem worldwide, mainly in tropical countries, including Brazil [1]. The Atlantic coast of Brazil is considered a biodiversity hotspot; however, the fragmentation of its ecosystems has a series of effects on its original vegetation and animal communities [2,3]. Therefore, hematophagous insects that have adaptive plasticity for the outskirts of cities and domiciliation may end up becoming serious pests, in addition to carriers of etiological agents of diseases to human and animal populations [4]. Studies of mosquito diversity in Atlantic Forest remnants, both in environments influenced by anthropogenic processes and in secondary growth fragments, are important to assess possible changes in behavior, distribution patterns, and activities of mosquito populations, including species previously considered exclusively sylvatic [5,6]. 

The main genera of mosquitoes capable of becoming infected and transmitting the sylvatic yellow fever virus (YFV) are *Haemagogus* Williston, 1896, and *Sabethes* Robineau-Desvoidy, 1827, and some species act as vectors in the natural cycle of this zoonosis in forested areas of the Americas. The *Haemagogus* spp. have a great diversity, including 28 species, nine of which have already been found in Brazil [7]. Some species from this genus have high epidemiological importance in the transmission of the sylvatic YFV [8]. 

The genus *Haemagogus* is restricted to the New World, and almost all species have a Neotropical distribution, except for *Haemagogus equinus* Theobald, 1903, which reaches some southern points of the Nearctic region [1]. They are essentially wild, diurnal, acrodendrophilic mosquitoes (insects that prefer to live/feed in the forest canopy) and primarily inhabit areas of dense forests and gallery forests [9,10,11,12,13,14]. 

Like other Aedinii mosquitoes, *Haemagogus* spp. tend to lay eggs in tree-trunk cavities, bamboo, tree hollows, and coconut shells [8]. The eggs are very resistant, present dormancy mechanisms, and commonly hatch during the rainiest time of the year, although each species can show different hatching patterns to stimuli by contact with water [7]. This strategy allows for the long-term survival of multivoltine mosquitoes that grow in temporary larval habitats and water containers subject to water fluctuations [15].

Two types of dormancy, quiescence and diapause, have been responsible for the evolutionary success of mosquitoes. Diapause, which is the suspension of egg development, involves a long and stable interruption of hatching, even when environmental conditions are favorable, allowing the egg to hatch even after an adverse condition. In contrast, the quiescence process is induced by unfavorable environmental conditions (temperature, desiccation, photoperiod, and others) and ceases soon after exposure to hatching stimuli, such as flooding [16]. However, some quiescent eggs may require more than one flood event to hatch, which is known as parcel hatching [17]. 

Oviposition choice, egg hatching, and development of immature larvae can also be affected by the presence of conspecific and heterospecific larvae in the same breeding site. Serpa et al. (2008) studied the effects of conspecific and heterospecific larvae in the same breeding site water and their influence on the oviposition of pregnant females; the authors found that *Aedes aegypti* laid more eggs when the larval rearing containers had *Ae. albopictus* larvae [18]. Such information can contribute to a better understanding of the ecological relationships between species, by evaluating the reproductive behavior of females in a situation of coexistence in a given breeding site [18].

Understanding species-specific patterns of the partial hatching of viable eggs can help predict peaks in mosquito abundance, species coexistence, and their potential risks in disease transmission, as well as set up appropriate mosquito control strategies [17,19]. Our study aimed to evaluate seasonal, local, and species-specific relationships in the mosquito sex ratio (male:female), multiple egg immersion, and Aedinii species co-occurrence in an Atlantic Forest fragment in the state of Rio de Janeiro, Brazil.

## 2. Materials and Methods

### 2.1. Ethics Statement

All research was carried out in accordance with scientific license number 44333 from the Ministry of the Environment (MMA), Chico Mendes Institute for Biodiversity Conservation (ICMBio), and Biodiversity Information and Authorization System (SISBIO). All members of the collection team were vaccinated against YFV and aware of the potential risks in the study areas.

### 2.2. Study Areas

The municipality of Casimiro de Abreu is 140 km from the city of Rio de Janeiro. The main vegetation cover in the region is characteristic of the Atlantic Forest biome, with dense submontane rainforests in moderate and advanced stages of regeneration. The region, located in the São João river basin, is in the intertropical zone (low latitudes).

**Figure 1:**
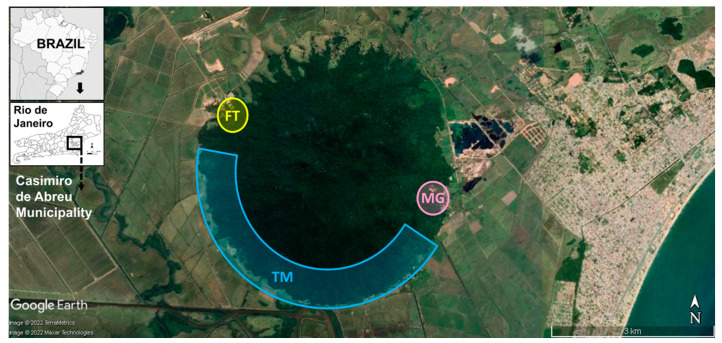
Location of three sampling sites—FT, MG, and TM—in an Atlantic Forest F = fragment at the municipality of Casimiro de Abreu, state of Rio de Janeiro, Brazil. Image adapted from Google Earth (accessed on 8 August 2022).

A fragment of the Atlantic Forest remnant in the municipality of Casimiro de Abreu was selected, due to its vulnerability to arbovirus transmission (Figure 1). The region was affected by a severe outbreak of yellow fever in 2016–2018 [20]; three sites were sampled in the forest because they were close to places where human transmission has been recorded. These sampling sites have different levels of preservation and legal status (two reserves and one private property), as follows: Três Montes Farm (FT), with an area of 194.0 ha (22°31′40.1″ S 42°02′58.6″ W); Três Morros private natural heritage reserve (TM), with an area of 508.78 ha (22°32′07.2″ S 42°03′18.9″ W); the privately-owned nature reserve Morro Grande (MG), with an area of 192.34 ha (22°32′37.2″ S 42°00′45.4″ W). According to the Köppen classification system, the climate is predominantly of the Aw type (Tropical wet-dry climate), with dry winters and humid summers, an average annual temperature of 24.5 °C, and average annual precipitation of 1200 mm [21]. 

### 2.3. Sampling Design and Egg Rearing

Sampling sites FT, MG, and TM were approximately 1 km apart. In each site, 6–7 trees were randomly selected for trap installation, and the minimum and maximum distances between them were from 80 m to 120 m.

Ovitraps were used to collect the eggs; oviposition containers consisted of a 300 mL matte black pot without a lid and with four plywood paddles (wooden paddles) measuring 2.5 by 14 cm, stuck vertically in the trap with paperclips. Natural water and litter were added to the pot, aiming to reproduce an ecosystem similar to the natural one [22]. The traps were monitored from August 2018 to December 2020, and the paddles inside the ovitraps were replaced monthly, identified according to the point, and transported to the Diptera Laboratory at Instituto Oswaldo Cruz.

In total, 50 ovitraps were randomly installed (FT, 15 traps; MG, 17 traps; TM, 18 traps) at different heights from ground level (0, 2, 4, 6, and 8 m). The ropes were thrown using a fishing lead of ≈4 cm in diameter, and hoisted using a nylon rope to install the traps in the trees. The positive paddles were separated in the laboratory, subjected to egg counting, and immersed in transparent trays containing type I ultrapure water. Subsequently, the trays containing the paddles were placed in a controlled experimental environment in a greenhouse with the temperature regulated at 28 ± 1 °C, relative humidity from 75 to 90%, and a photoperiod of 12 h. After three days, the paddles were removed from the water and allowed to dry at room temperature for another three days. During paddle removal, the number of hatched larvae was recorded; the immature ones were kept alive according to the protocol of Alencar et al. (2008), aiming at identification at the species level when reaching adulthood. The eggs in the paddles were subjected to repeated immersion and drying cycles, until all had hatched [23].

The mosquito species were identified by direct observation of the morphological characters evident under an optical microscope (Leica DMD108^®^), using dichotomous keys proposed by Arnell (1973), Forattini (2002), and Marcondes and Alencar (2010) [7,8,24]. The abbreviations of generic and sub-generic names follow Reinert (2009) [25]. After the species identification, all specimens were deposited in the entomological collection of the Oswaldo Cruz Institute, Fiocruz, under the title “Atlantic Forest Mosquito Collection, Rio de Janeiro”.

### 2.4. Statistical Analysis

Generalized linear mixed models (GLMM) were used to compare the mosquito sex ratios (male:female) between seasons (rainy and dry) and forest sites (FT, MG, and TM) as fixed factors. “Month” (for each season) and “tree” (for each site) were set as random effects in the models, and probable temporal and spatial autocorrelations in the outcomes were considered. The models were fitted to a binomial error distribution (see Crawley 2005) using the package *glmmTMB*, due to its flexibility for dealing with over- and underdispersed data and excess of zero scores [26,27].

Model adequacy was verified utilizing residual diagnostics (distribution, dispersion, and outliers); when necessary, model residuals were checked for temporal autocorrelation using the Durbin–Watson (DW) test and ACF plot inspection (*DHARMa* package 0.4.5). The coefficient estimation outputs were automatically back-transformed (exponential) and tabulated using *sjPlot* 2.8.4 and *sjmisc* 2.8.5. (Supplementary File S1). All graphs and analyses were performed in the R Platform version 3.6.0 [28].

## 3. Results

A total of 33,091 eggs were counted in the paddles of ovitraps installed in the three sites of the Atlantic Forest remnant, of which about 18% were already hatched (6152 eggs), and 26,939 were intact and not hatched. The paddles were subjected to immersion events for egg hatching, and larvae were reared until the emergence of adults. Of the total number of non-hatched eggs, 20,461 larvae were obtained, representing a hatching rate of approximately 76% (Table 1). However, the proportion of medically important adults from the collected eggs was 25% (n = 5192), for which four species of epidemiological importance were identified: *Aedes albopictus* (n = 1277), *Ae. terrens* (n = 793), *Haemagogus janthinomys* (n = 89) and *Hg. leucocelaenus* (n = 3033).

### 3.1. Sexual Proportion x Site and Season

**Figure 2:**
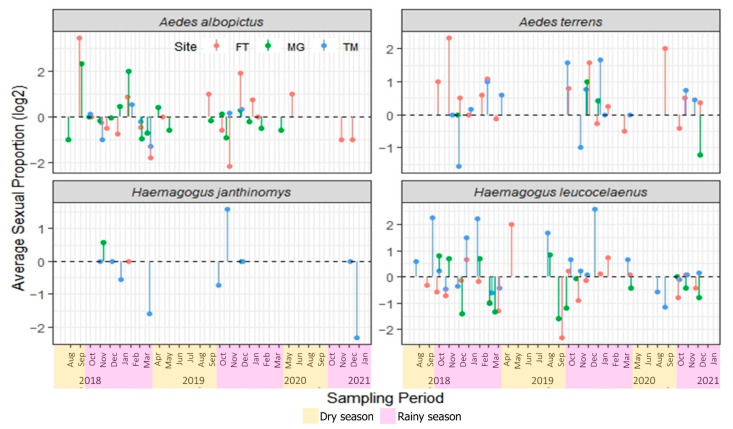
Sexual proportion of medically important mosquito species from August/2018 to January/2021 in three sampling sites at an Atlantic Forest fragment in Casimiro de Abreu, Rio de Janeiro, Brazil. Values were logarithmic-transformed (log_2_) for visual purposes only. Black dashed line represents the male:female proportion equal to 1.

Considering the total number of adults that emerged in the laboratory (n = 5192), the number of females (50.3%) and males (49.7%) was similar, regardless of the collection site, sampling month, and species. However, the sex ratio (male:female) varied greatly depending on the sampling sites and seasons, without, however, depicting a clear pattern for the species (Figure 2, Table 2).

The GLMM estimates found no difference in the variation of the average sex ratio as a function of these predictors for most species. However, this ratio significantly differed between the FT and TM sites for *Hg. leucocelaenus* (estimate, 1.40; CI, 1.07–1.84). As for random effects, the sample design based on a continuous time series contributed a small part of the variability in the average sex ratio recorded for *Ae. albopictus* (τ00 Season: Month = 0.25) and *Hg. leucocelaenus* (τ00 Season: Month = 0.21); however, there was no temporal dependence effect on the variability of these species (*Ae. albopictus*: DW test = 2.4717, *p*-value = 0.2259; *Hg. leucocelaenus*: DW = 2.1949, *p*-value = 0.621, Supplementary File S1).

Sex ratio: male:female proportion considering the sum of individuals per site or season. *Statistically significant difference from the basal level of the “Site” predictor, the FT site (see Supplementary File S1). In general, the proportion of males and females did not differ between sites and seasons for all mosquito species.

### 3.2. Egg Immersion Event x Mosquito Species x Sex

**Figure 3:**
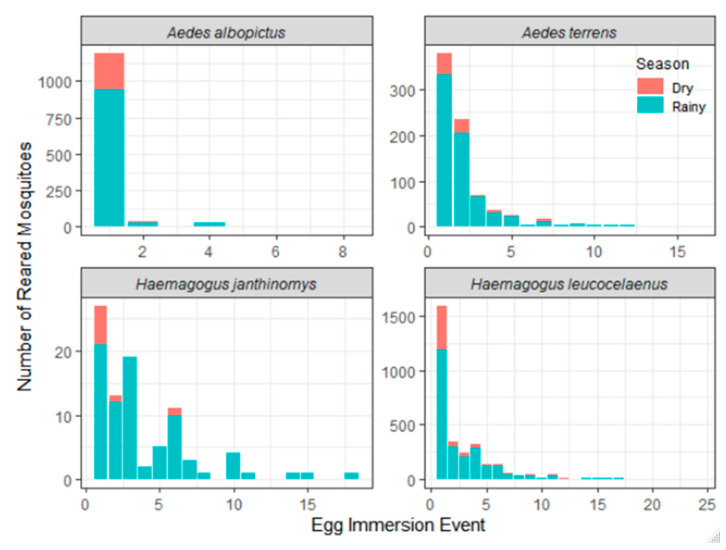
Abundance of adult vector mosquitoes per egg immersion event during the dry (red) and rainy (blue) seasons in Casimiro de Abreu, Rio de Janeiro, Brazil.

The number of immersions required for hatching eggs adhered to the paddles differed between mosquito species. For *Ae. albopictus*, the first immersion event was the most successful in reaching the adult stage; the sixth, seventh, and eighth events produced negligible numbers of mosquitoes. In turn, most of the eggs of typically wild species (*Ae. terrens*, *Hg. leucocelaenus*, and *Hg. janthinomys*) were in diapause or quiescence, and needed more than one sequential immersion event for their outbreak. In both the dry and rainy seasons, eggs seemed to hatch in the first and subsequent events (Figure 3).

**Figure 4:**
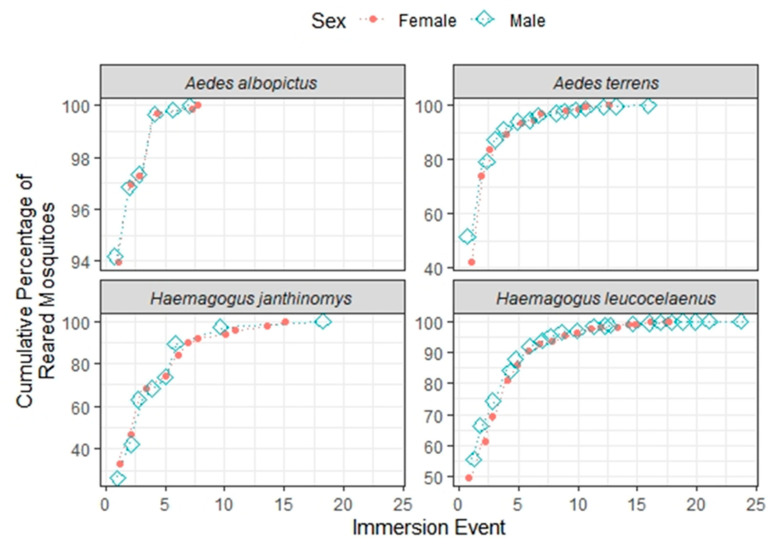
Cumulative percentages of the number of eggs hatched by sex throughout the immersion events for the mosquito species *Ae. albopictus*, *Ae. terrens*, *Hg. janthinomys*, and *Hg. leucocelaenus*.

Regarding the comparison between male and female mosquito hatching along the immersion events, two general tendencies were observed: (1) male mosquitoes of typically sylvatic species (and less prone to adaptation) usually took longer to emerge, and (2) and they required more immersion events, which was more evident for males of *Hg. leucocelaenus* and *Ae. terrens* (Figure 4).

### 3.3. Co-Occurrence of Mosquito Species

**Figure 5:**
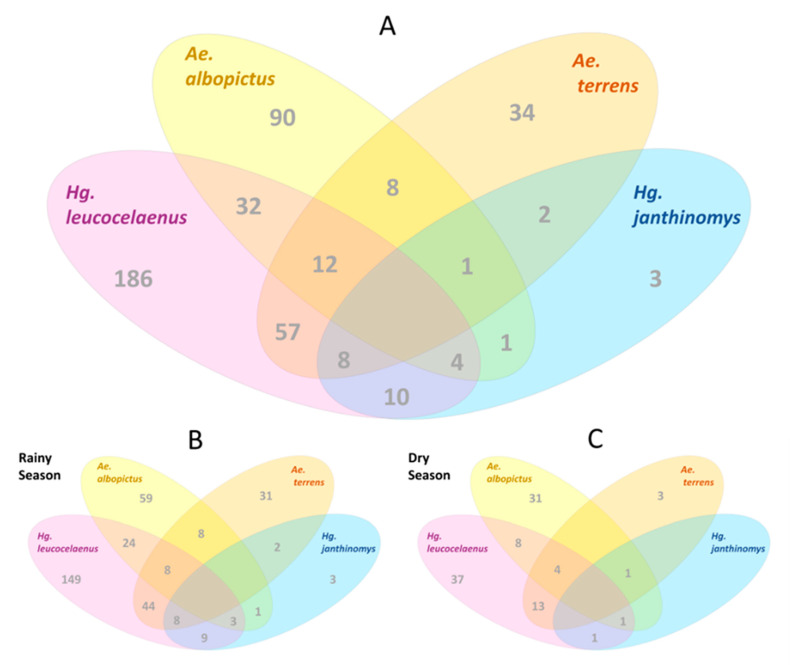
Co-occurrence of mosquito species in ovitraps placed in an Atlantic Forest fragment (**A**) during rainy (**B**) and dry seasons (**C**) in Casimiro de Abreu, Rio de Janeiro, Brazil.

Paddles were negative for 884 of the total number of installed ovitraps. Of the 448 positive paddles for Aedinii eggs, 70% gave rise to adult mosquitoes of the same species (313 paddles). Considering the events of species co-occurrence in the same trap (Figure 5A), the most frequently observed combination was the co-occurrence of *Ae. terrens* and *Hg. leucocelaenus* (n = 57), followed by *Ae. albopictus* and *Hg. leucocelaenus* (n = 32), *Ae. albopictus*, *Ae. terrens*, and *Hg. leucocelaenus* (n = 12), and *Hg. janthinomys* and *Hg. leucocelaenus* (n = 10). However, the frequency of these interactions differed between seasons, as did the number of positive paddles and egg density per season. In the rainy season, 349 paddles had successful egg hatching (Figure 5B) compared to only 99 in the dry season (Figure 5C). Contrary to expectations, co-occurrence events were less frequent in the dry season. We first supposed there would be less availability of natural breeding sites in the dry season, and thus the competition for breeding sites would be greater, compared to the rainy season. On the other hand, in the dry period, populations of wild mosquitoes occurred at low densities, and the record of co-occurrence depended on greater sampling effort during this period.

## 4. Discussion

Studies on the distribution patterns and activities of mosquito populations in remnants of the Atlantic Forest are of paramount importance, and are influenced by anthropogenic activities and preserved fragments of secondary growth. Events such as fragmentation of forest areas, rapid expansion of urban settings, poor sanitary conditions aligned with climate change, and the pollution of natural environments, directly influence the proliferation and spread of mosquito vectors and arbovirus transmission [29,30]. Vector mosquitoes from the genera *Aedes* and *Haemagogus* are commonly found in Atlantic Forest fragments, and have been described in various natural parks and environmental protection areas in Rio de Janeiro, southeastern Brazil [31,32,33].

Four Aedinii species were collected in our study: *Ae. albopictus*, *Ae. terrens*, *Hg. leucocelaenus*, and *Hg. janthinomys*. The anthropophagic and opportunistic *Ae. albopictus*, which was the second most abundant species in our study, has been frequently found in all gradients across forest, rural, and urban areas [34]; in contrast, the acrodendrophilic *Ae. terrens* has a generalist oviposition behavior, laying eggs both in natural and artificial containers. However, the blood sources and host preferences of this sylvatic culicid are still unknown [13,35].

*Haemagogus leucocelaenus,* the most common and numerous species sampled in our study, is considered an acrodendrophilic species; however, its presence was recorded in human dwellings and secondary vegetation. A study carried out in Caxiuanã National Forest, Pará, Brazil, confirmed its diurnal habit, between 2 pm and 3 pm, showing that its predominance in the soil and/or forest canopy depends on the season and month of the year [36]. In turn, *Hg. janthinomys* is thought to be less likely to adapt to microclimatic and environmental changes including its oviposition behavior [37,38], although recent findings demonstrate that it can also be found on the forest ground and at the edges and in open fields [38].

Mosquito sex-ratio studies have led to some interesting findings; in 1966, Hickey and Craig mentioned that the male parent determines the sex ratio in the progeny and, given normal segregation, equal numbers of males and females should occur [39]. We found that the number of females (50.3%) and males (49.7%) was similar, considering the total number of adults reared in the laboratory (n = 5192). Lounibos and Escher (2008) observed that *Ae. albopictus* from Florida, USA, showed a significant bias in the number of male specimens [40]. Their results are in line with the ones of the present study, as we found greater amplitudes of variation for *Ae. albopictus*, with the sex ratio also biased toward males. A bias in the number of male specimens was also observed for *Hg. leucocelaenus*; however, no evidence was found in the literature to support or refute this observation, emphasizing the importance of further studies with this approach in the field of medical entomology.

In our study, this difference in sex ratio was observed in mosquito eggs collected from forest fragments, and therefore, from mosquitoes occurring naturally in these forest fragments. A male-skewed ratio may be a positive event, since male-biased reproductive sex ratios have previously been suggested as an attractive method to suppress or eliminate pest populations, obviously on a much larger scale [41].

Most of the eggs from sylvatic mosquito species were in diapause and required more than one sequential immersion event for their hatching. This is very commonly observed in *Haemagogus* species [42]. Considering the absolute values of eggs hatched, *Hg. leucocelaenus* eggs hatched until the 21st immersion. A similar result was observed by Silva et al. (2018), where eggs from this species showed installment hatching up to the 37th immersion. Moreover, hatching rates for this species were 1.5 times higher in the rainy season than in the dry season [43].

Mosquito species from the studied forest remnant diverged in the number of immersions required for egg hatching. For *Ae. albopictus*, the first immersion event resulted in the most successful egg hatching. These results agree with other studies that also show that eggs from this species tend to hatch on first immersion, regardless of the season. More immersion events might be required for hatching eggs sampled closer to the dry winter season as part of a strategy to ensure egg viability and further larval development [42].

A higher number of collected and hatched eggs from *Hg. leucocelaenus* has also been observed in other fragments of the Atlantic Forest, in which eggs were continuously collected throughout the study and also the most frequently captured species [44,45,46]. It is important to note that the collection methodology used in this study and the location where the mosquitoes were captured will invariably select species with wild habits, and those that lay their eggs on the water surface.

Our findings suggest that typically wild species are less prone to adaptation, and require more immersion events; this was more evident for males, while the proportion of males and females varied greatly, according to sampling sites and seasons, but without a clear pattern for the species. Contrary to expectations, events of co-occurrence of mosquito species were less frequent in the dry season. Further studies on multiple egg emersion could delve deeper into the effects of environmental and genetic components and their possible interaction, to understand the mechanisms behind the biological adaptations of Aedinii eggs.

Ecological relationships between *Hg. leucocelaenus*, *Hg. janthinomys*, *Ae. terrens*, and *Ae. albopictus* have been previously described. Forest vertical stratification, meteorological and seasonal effects, and coexistence have been extensively explored for these vector species [13,22,31,32]. *Haemagogus leucocelaenus* and *Hg. janthinomys* showed a strong correlation between the number of overlapping eggs for the same breeding sites and the same paddles [33]. Species of other genera, such as *Limatus*, *Culex*, *Wyeomyia*, and *Toxorhynchites*, oviposit in forest water ovitraps, and their coexistence deserves further investigation [14].

## 5. Conclusions

The constant change in the epidemiology of zoonotic viruses can be attributed to the spreading of the virus to new areas, which happens through hosts and vectors. Studies on vector mosquito oviposition in forest habitats are rare, especially those that assess the coexistence of sylvatic mosquito species, their breeding sites, habitat preferences, oviposition behavior, site selection, and density-dependent competition. The present study increases the available information on the sex ratio of epidemiologically relevant mosquito species from *Haemagogus* and *Aedes* in nature, along with the co-occurrence of important vector species from these genera, such as the yellow-fever-virus vector *Hg. leucocelaenus*, and *Ae. albopictus,* a secondary vector of dengue, Zika, and yellow-fever viruses. Considering these viruses are endemic to Brazil and are a burden on the human population, studies such as this help shed some light on how these vectors behave in nature, and can be used by mosquito surveillance programs, such as the “Plano de Contingência para Enfrentamento às Arboviroses” (the Contingency Plan to Combat Arboviruses).

## Figures and Tables

**Table 1:** Number of hatched and unhatched eggs, larvae/adult proportions, and reared adult mosquitoes per sampling site at an Atlantic Forest remnant in Casimiro de Abreu, Rio de Janeiro, Brazil.

Parameters	Sites		
	FT	MG	TM
Hatched eggs	1850	1856	2446
Unhatched eggs	10,003	5338	11,598
Proportion of larvae/unhatched eggs	0.69	0.49	0.58
Proportion of adults/larvae	0.34	0.39	0.34
**Reared adults**			
*Aedes albopictus*	519	578	180
*Aedes terrens*	414	35	344
*Haemagogus leucocelaenus*	1068	445	1520
*Haemagogus janthinomys*	8	8	73

**Table 2:** Male and female mosquitoes and sex ratio per mosquito species, season, and sites (FT, MG, and TM) sampled using ovitraps installed at an Atlantic Forest remnant, Casimiro de Abreu, Rio de Janeiro, Brazil.

Species	N	Site			Season	
		FT	MG	TM	Dry	Rainy
*Aedes albopictus*	Female	260	325	91	130	546
	Male	259	253	89	126	475
	**Sex ratio**	1.00	0.78	0.98	0.97	0.87
*Aedes terrens*	Female	185	16	133	46	288
	Male	229	19	211	52	407
	**Sex ratio**	1.24	1.19	1.59	1.13	1.41
*Haemagogus janthinomys*	Female	5	3	43	6	45
	Male	3	5	30	2	36
	**Sex ratio**	0.60	1.67	0.70	0.33	0.80
*Haemagogus leucocelaenus*	Female	589	233	728	309	1241
	Male	479	212	792	273	1210
	**Sex ratio**	0.81	0.91	1.09*	0.88	0.98

## Data Availability

Not applicable.

## Supplementary Materials

The following supporting information can be downloaded at: https://www.mdpi.com/article/10.3390/life13010013/s1. Supplementary File S1. Summary of generalized linear mixed models (GLMM) to explain the proportion of male:female mosquitoes as a response to local and seasonal variations in three sites at an Atlantic Forest remnant, Casimiro de Abreu, state of Rio de Janeiro, Brazil.
