# Supplementary material for: Sexual Proportion and Egg Hatching of Vector Mosquitos in an Atlantic Forest Fragment in Rio de Janeiro, Brazil
Source: Life (Basel). 2022 Dec 21;13(1):13. doi: 10.3390/life13010013 (PMC9912254; doi:10.3390/life13010013)
Supplement: Supplementary file 1 [file life-13-00013-s001.zip › life-2008349 Supplementary.pdf]

Supplementary File S1. Summary of generalized linear mixed models (GLMM) to explain the proportion of male:female mosquitoes as a response to local and seasonal variations in three sites at an Atlantic Forest remnant, Casimiro de Abreu, state of Rio de Janeiro, Brazil.

Model adequacy was assessed by using the 'DHARMa' package plots (residual diagnostics: distribution, dispersion and outliers) and estimation output was tabulated using sjPlot 2.8.4 and sjmisc 2.8.5. All graphs and analyses were performed in the R Plataform version 3.6.0 (R Core Team, 2019).

Table S1. Results from generalized linear mixed models (GLMM) testing the effects of fixed (Seasons and Sites) and random (Months and Trees) factors on the proportion of males:females for each mosquito species.

Model Term	***Aedes albopictus***			***Aedes terrens***		
	Estimate	CI	p	Estimate	CI	p
**Fixed effects**						
Intercept*	1.49	0.82 – 2.69	0.191	1.01	0.65 – 1.57	0.978
Site MG	0.99	0.68 – 1.45	0.973	0.94	0.46 – 1.95	0.874
Site TM	1.19	0.74 – 1.91	0.476	1.32	0.92 – 1.88	0.129
Rainy Season	0.60	0.32 – 1.11	0.103	1.26	0.81 – 1.94	0.303
**Random Effects**						
σ2	3.29			3.29		
$\tau 00_{\text{Site:Tree}}$	0.03			0.02		
$\tau 00_{\text{Season:Month}}$	0.25			0.00		
ICC	0.08			-		
N_{Site}	3			3		
N_{Tree}	19			16		
N_{Season}	2			2		
N_{Month}	25			19		
Observations	148			122		
	Haemagogus janthinomys			***Haemagogus leucocelaenus***		
**Fixed effects**	Estimates	CI	p	Estimates	CI	p
Intercept*	0.26	0.03 – 2.39	0.236	0.93	0.60 – 1.45	0.753
Site MG	2.78	0.37 – 21.03	0.323	1.09	0.79 – 1.49	0.611
Site TM	1.26	0.28 – 5.73	0.764	1.40	1.07 – 1.84	**0.014**
Rainy Season	2.27	0.43 – 12.11	0.337	0.86	0.53 – 1.38	0.528
**Random Effects**						
σ2	3.29			3.29		
$\tau 00_{\text{Site:Tree}}$	0.00			0.03		
$\tau 00_{\text{Season:Month}}$	0.00			0.21		
ICC	-			0.07		
N_{Site}	3			3		
N_{Tree}	8			19		
N_{Season}	2			2		
N_{Month}	11			25		
Observations	29			309		

MODEL RESIDUAL DIAGNOSTICS – DHARMa (Hartig 2020)

For each mosquito species, models were computed using the male:female proportion data as response variable, and seasons (Dry and Rainy seasons) and sampling sites (FT, MG, and TM) as predictors, fitted to a binomial error distribution. Two random factors have been considered in the models, sampling months in each season and trees in each site. Months within seasons were included in the model as random factor to minimize the effect of temporal autocorrelation in the trap outcomings. Trees in the same site were considered as potential source of spatial autocorrelation.

Figure S1. Residual diagnostic plots for the binomial GLMMs. Models were computed for male:female proportions of four mosquito species sampled at an Atlantic Forest remnant, Casimiro de Abreu, state of Rio de Janeiro, Brazil.

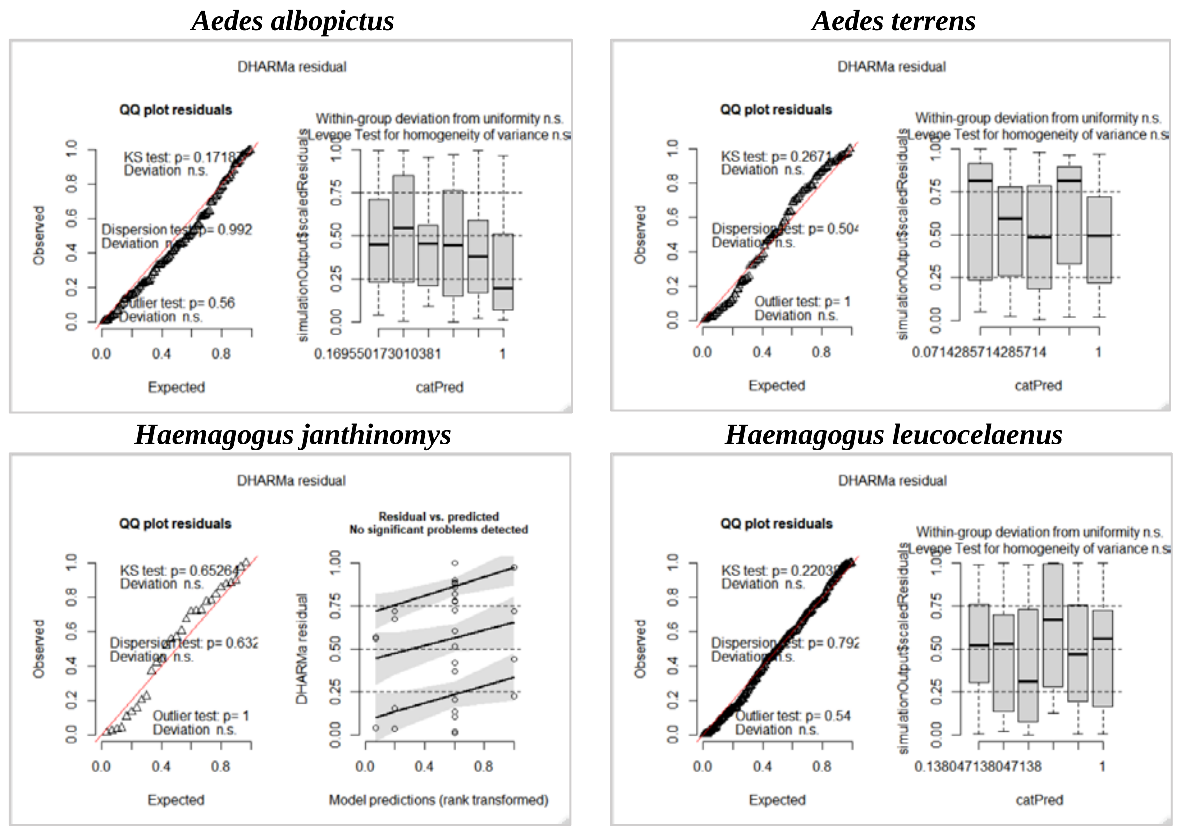

CHECKING FOR TEMPORAL AUTOCORRELATION

***Aedes albopictus* - Durbin-Watson test**

data: simulationOutput$scaledResiduals ~ 1

DW = 2.4717, p-value = 0.2259

alternative hypothesis: true autocorrelation is not 0

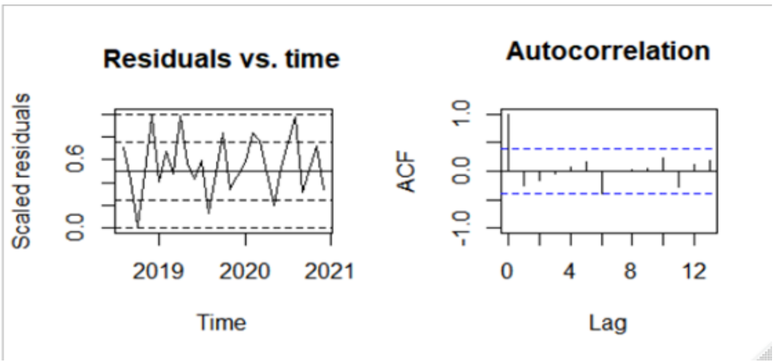

Haemagogus leucocelaenus **- Durbin-Watson test**

data: simulationOutput$scaledResiduals ~ 1

DW = 2.1949, p-value = 0.621

alternative hypothesis: true autocorrelation is not 0

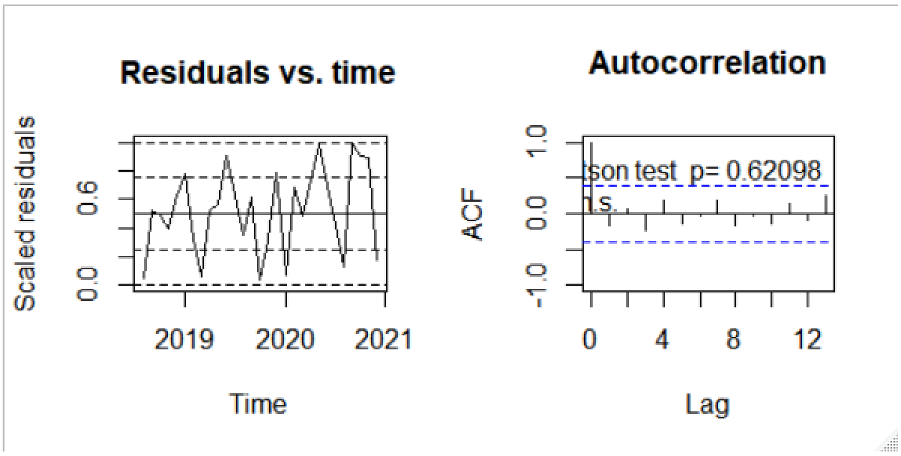

References

Hartig F. DHARMa: residual diagnostics for hierarchical (multi-level/mixed) regression models. 2022-01-16. https://cran.r-project.org/web/packages/DHARMa/vignettes/DHARMa.html

R Development Core Team, R: a language and environment for statistical computing. Version 3.6.1 (2019). R Foundation for Statistical Computing, Vienna, https://www.R-project.org, [accessed 18 December 2019]
